# Live while the DNA lasts. The role of autophagy in DNA loss and survival of diploid yeast cells during chronological aging

**DOI:** 10.18632/aging.205102

**Published:** 2023-10-09

**Authors:** Tuguldur Enkhbaatar, Marek Skoneczny, Karolina Stępień, Mateusz Mołoń, Adrianna Skoneczna

**Affiliations:** 1 Institute of Biochemistry and Biophysics, Polish Academy of Sciences, Warsaw 02-106, Poland; 2 Institute of Medical Sciences, Rzeszów University, Rzeszów 35-959, Poland; 3 Institute of Biology, Rzeszów University, Rzeszów 35-601, Poland

**Keywords:** aging, genome instability, lifespan, autophagy, double-strand breaks

## Abstract

Aging is inevitable and affects all cell types, thus yeast cells are often used as a model in aging studies. There are two approaches to studying aging in yeast: replicative aging, which describes the proliferative potential of cells, and chronological aging, which is used for studying post-mitotic cells. While analyzing the chronological lifespan (CLS) of diploid *Saccharomyces cerevisiae* cells, we discovered a remarkable phenomenon: ploidy reduction during aging progression. To uncover the mechanism behind this unusual process we used yeast strains undergoing a CLS assay, looking for various aging parameters. Cell mortality, regrowth ability, autophagy induction and cellular DNA content measurements indicated that during the CLS assay, dying cells lost their DNA, and only diploids survived. We demonstrated that autophagy was responsible for the gradual loss of DNA. The nucleophagy marker activation at the start of the CLS experiment correlated with the significant drop in cell viability. The activation of piecemeal microautophagy of nucleus (PMN) markers appeared to accompany the chronological aging process until the end. Our findings emphasize the significance of maintaining at least one intact copy of the genome for the survival of post-mitotic diploid cells. During chronological aging, cellular components, including DNA, are exposed to increasing stress, leading to DNA damage and fragmentation in aging cells. We propose that PMN-dependent clearance of damaged DNA from the nucleus helps prevent genome rearrangements. However, as long as one copy of the genome can be rebuilt, cells can still survive.

## INTRODUCTION

Aging is an inevitable process that affects all organisms, regardless of whether they are unicellular or part of higher-organized multicellular organisms, such as mammals. This process is of great interest to researchers, as each new study in this area brings hope for discovering ways to delay aging. Since aging is a universal cellular problem, it is understandable that yeast cells are commonly used as a model for studying aging (reviewed in [1, 2]). Two main approaches to studying aging in yeast cells are used [3, 4], and these approaches yield different lifespan parameters [5]. Replicative lifespan (RLS) refers to the number of mitotic divisions a given mother cell can undergo before reaching senescence and death [6]. Senescence is the period after the last division during which the cell is still alive [5]. Chronological lifespan (CLS) refers to the length of time a post-mitotic cell population (in yeast, stationary phase culture) can still give rise to progeny [7].

We have learned a great deal about the factors that influence aging, some of which are related to mutations in the genome. It is well known that RLS is strongly influenced by signal transduction pathways’ efficiency (such as TOR [8] or PKA-dependent pathways [9]), chromatin remodeling ([10], with sirtuins [11–13] playing a widely explored role), DNA damage response pathways’ effectiveness in preventing genome instability and ensuring proper DNA segregation [14, 15], metabolic potential of the cells [16–19], and cell polarity and size [20–22]. A growing body of evidence suggests that CLS depends on similar factors. Signal transduction pathways, chromatin remodeling, and genome maintenance [7, 11, 23, 24], proper stress response [25], metabolic potential of the cells [26–30], and cell wall content, which determines stress resistance and cell size [31], are all crucial for CLS. Yeast cells lacking mitochondrial or autophagy-related genes in their genomes consistently exhibit a shortened CLS [32]. Lifespan also depends on extracellular factors; for example, glucose limitations cause lifespan extension [33]. This effect, known as caloric restriction, can be nullified by methionine supplementation [34]. Similarly, mutations that affect signal transduction pathways can lead to improper sensing of environmental resources [35], resulting in similar outcomes. However, despite identifying some mutations in the cellular background or environmental conditions that might influence aging speed, we still do not know the real factor that limits/determines cell lifespan.

In our previous research, we investigated whether disturbances in the G1/S phase transition, such as a delay in the START point of replication due to reduced levels of proteins required to initiate this process, could affect the CLS of yeast. Using diploid *Saccharomyces cerevisiae* strains heterozygous for genes encoding subunits of the origin recognition complex (*ORC1* to *ORC6*), we found that reducing the levels of any of the Orc1–6 proteins resulted in a significant increase in the budding lifespan and delayed average chronological aging, likely due to the delay in the cell cycle and subsequent extension of the doubling time [36]. During this study, we observed unexpected phenotypes of *S. cerevisiae* cells. At some stage in CLS, diploid cells reduced their DNA content to the level typical of haploid cells. We ruled out sporulation as the cause of haploidization, but how it occurs remained unanswered. In this paper, we attempted to address this issue. We demonstrated that a decrease in DNA content is accompanied by activating two autophagy pathways: nucleophagy and PMN. We also found that cells require at least one intact genome to survive the CLS experiment since cells with less than this minimum were dying, both haploids and diploids. In other words, cells live as long as their genome is restorable, and full DNA content is the limit of life.

## RESULTS

### Cells losing their DNA during CLS

**Figure 1:**
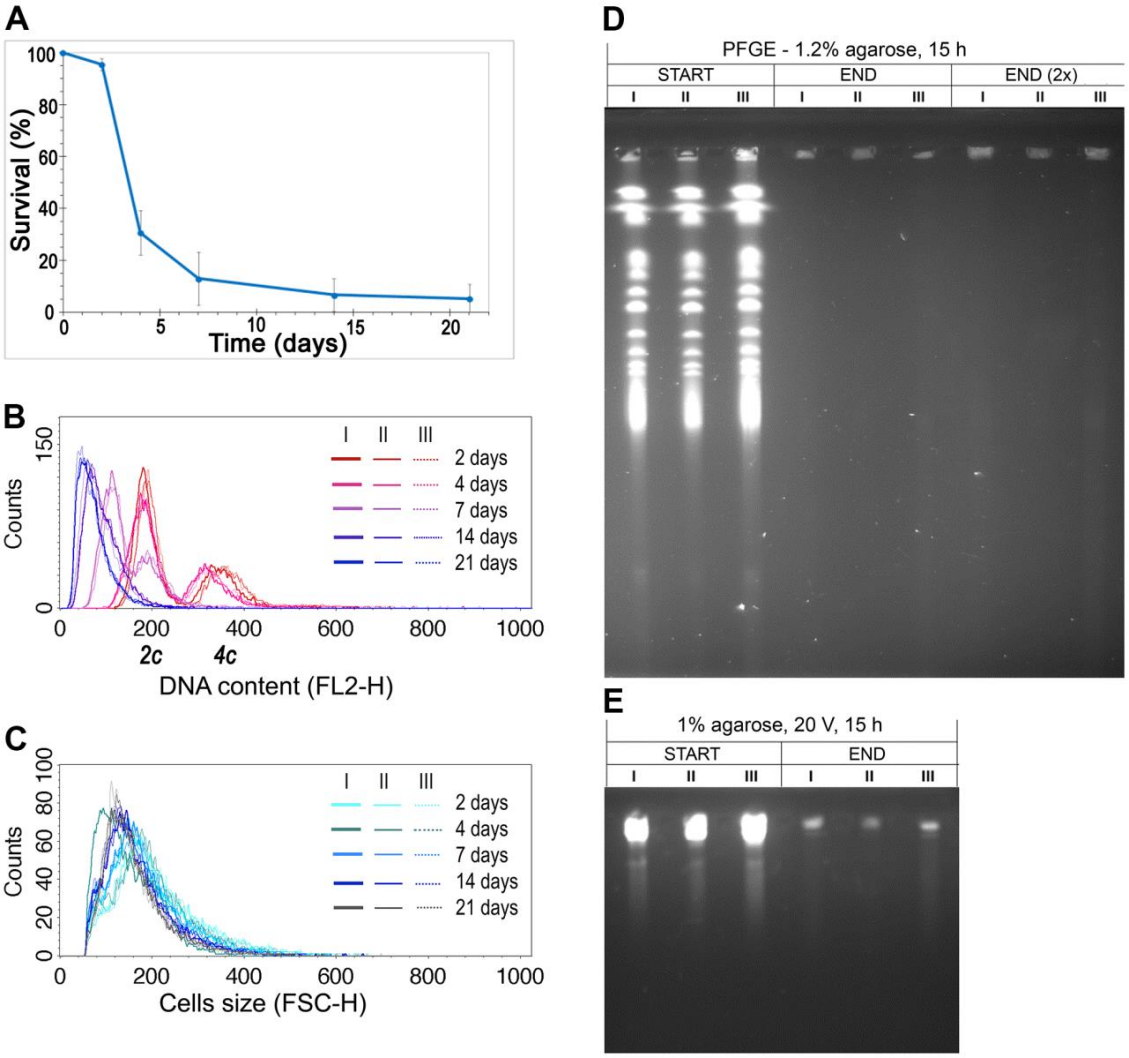
**DNA content reduction is associated with the CLS of the diploid yeast.** (**A**) CLS of diploid (BY4743) wild-type strain. The mean from three biological repetitions is shown. Bars indicate standard deviations. (**B**) Changes in the DNA content of the WT (BY4743) strain during CLS were assessed by flow cytometry of propidium iodide-stained cells. Three biological repetitions labeled I, II, and III are shown. *2c*, two DNA content (DNA content typical for diploid in G1 phase), *4c*, four DNA content (DNA content typical for diploid in G2 phase of the cell cycle) (**C**) The cell size changes observed in the WT (BY4743) strain during CLS assessed by flow cytometry. Three biological repetitions are shown. (**D**, **E**) The genome integrity of yeast cells, subject to CLS, at the 2^nd^ day (START) and 21^st^ day (END) of the experiment. Chromosome integrity was assessed by PFGE (**D**) and classical agarose electrophoresis (**E**). Three biological repetitions were analyzed. END (2×), for better DNA visualization, the amount of analyzed sample was doubled.

The data we obtained earlier drew our attention to the fact that diploid yeast cells analyzed during CLS had a reduced DNA content [36]. This phenomenon was observed not only in cells of six heterozygous strains with decreased expression of respective ORC complex subunits but also in the control strain, in all biological repetitions. The result was surprising but repetitive, and certainly worth further investigation. To validate this observation, we repeated the CLS experiment once more using a diploid wild-type strain, and we observed the same result (Figure 1A–1C; see also Supplementary Figure 1 for the controls). During the CLS assay, cell viability significantly decreased, and with time, diploid *S. cerevisiae* cells reduced their ploidy to the typical haploid level while their size remained relatively constant.

We considered several hypotheses to explain the ploidy reduction observed during CLS, including (1) sporulation, (2) DNA degradation, and (3) gradual loss of one copy of the genome. It is also possible that the apparent ploidy reduction is due to DNA condensation, which reduces its saturation with propidium iodide (PI), resulting in no change in the DNA content of aging cells. However, we excluded the hypothesis of sporulation as a source of ploidy reduction based on several arguments. First, a microscopic examination of the strain cultures during the CLS assay revealed no tetrads. Second, spores cannot germinate under nutrient-poor and inaccessible conditions. To verify the hypothesis of DNA degradation as a cause of DNA content reduction, we performed pulse-field gel electrophoresis (PFGE) to analyze the integrity of yeast chromosomes at the beginning and end of the CLS assay (Figure 1D). Our results showed that DNA in the aged cells undergoes fragmentation (Figure 1E). However, the histogram of DNA content we observed in flow cytometry analysis (Figure 1B) did not show DNA debris that should appear as a peak close to the Y-axis. We considered that a substantial part of DNA fragments might be lost during the lengthy preparation of DNA embedded in agarose plugs before PFGE analysis. With these conflicting results, we could not determine whether the gradual loss of DNA or DNA degradation is the source of ploidy reduction during CLS. Nevertheless, we could rule out the possibility that genomic DNA condenses over time, making fluorescent dye intercalation difficult and causing an artificial ploidy shift.

### Cells surviving CLS are diploids

**Figure 2:**
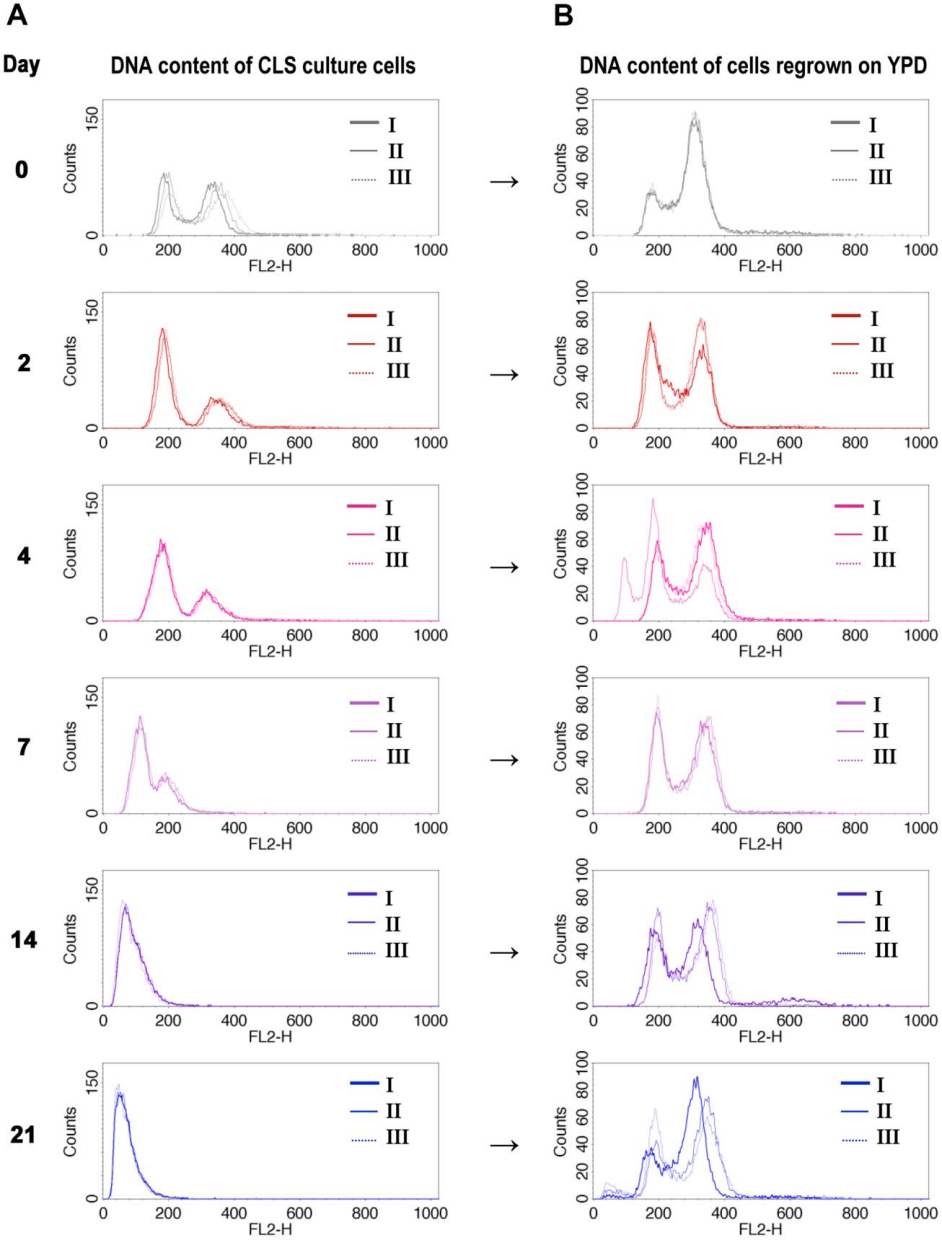
**DNA content of cells that survived CLS assay is diploidal.** DNA content of wild-type diploid cells (BY4743) during CLS showed ploidy reduction (**A**), but only diploids (or near-diploids) can survive and start to grow when transferred to the fresh medium (**B**). The I, II, III labels indicate the biological repetition.

As the previous results did not provide a clear answer to the question of the mechanism behind the observed ploidy shift during CLS of diploid yeast, we took a different approach. We asked what was the ploidy of cells that remained alive at each time point of the CLS experiment. To answer this question, we sampled the aging cells at the specific days of the CLS experiment and inoculated them into fresh medium. Then we compared the DNA content of the aging cells with the DNA content of regrown cells from the same day (see Materials and Methods for more details). Figure 2A, 2B display the results of this experiment, which showed that only diploid cells could regrow.

### A single copy of the genome allows surviving CLS of both haploids and diploids

**Figure 3:**
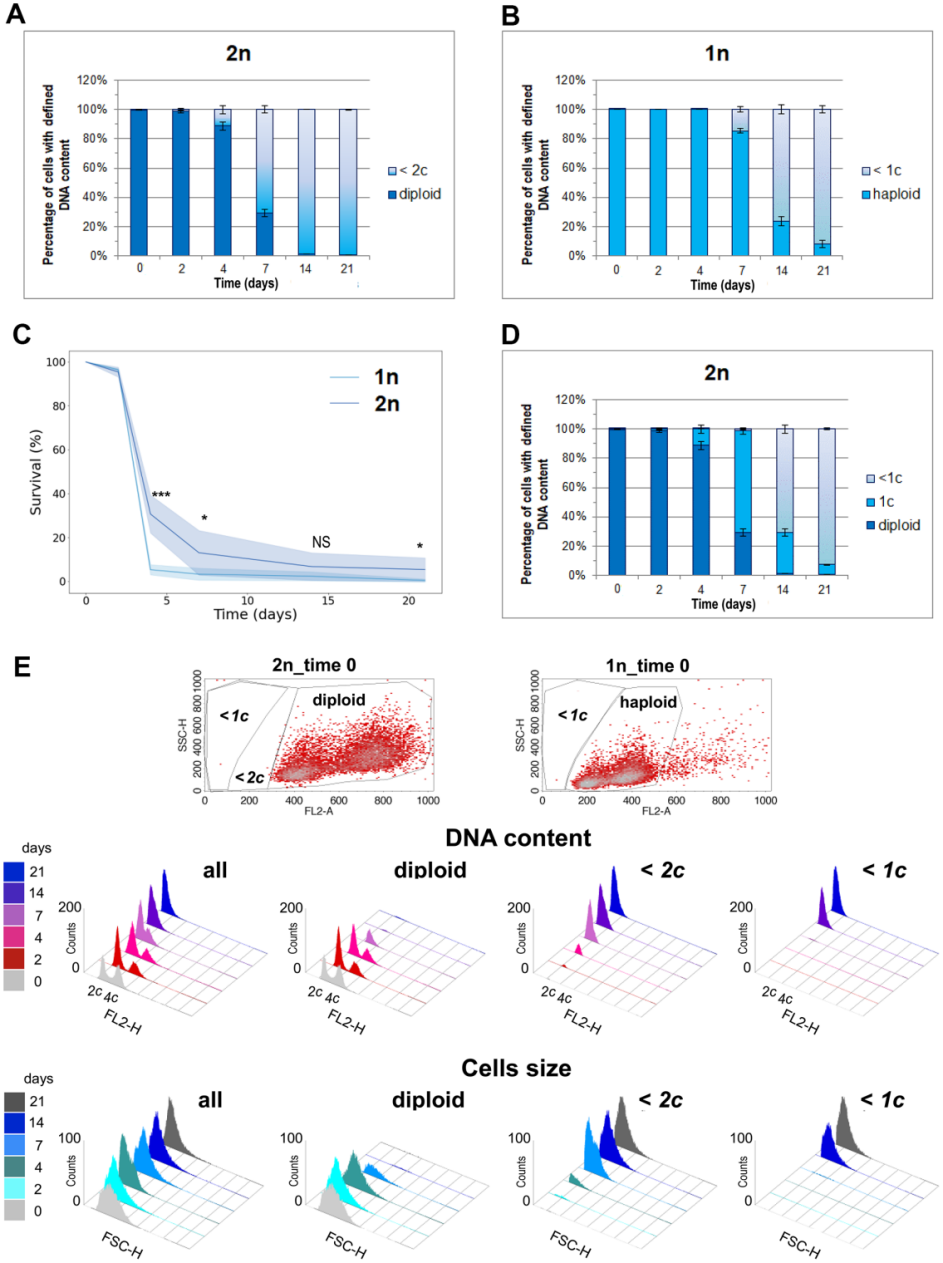
**One DNA content permits the survival of the CLS assay.** (**A**) The number of cells with diploid and lower than *2c* DNA content was observed at different time points during CLS of the diploid (BY4743) strain. (**B**) Number of cells with haploid and lower than *1c* DNA content observed in different time points during CLS of haploid (BY4741) strain. (**C**) Comparison of survival rate of diploid (BY4743) versus haploid (BY4741) wild-type strains during CLS assay. The mean from three biological repetitions is shown. Bars indicate standard deviations. Statistical significance was assessed using ANOVA and Dunnett’s post hoc test (* *p* < 0.05; *** *p* < 0.001). (**D**) Number of cells with diploid, *1c*, and lower than *1c* DNA content observed in different time points during CLS of diploid (BY4743) strain. (**A**, **C**, **D**) The mean from three biological repetitions is shown. Bars indicate standard deviations. (**E**) Flow cytometry results show changes in the DNA content and cell size during the CLS experiment in the whole cell population and its fractions defined as diploids and cells with DNA contents lower than *2c* or *1c*. The upper panel shows scatter plots for exponentially grown haploid and diploid strains, which enabled the setting up of gating conditions to determine specific subpopulations.

Since only diploids survived in the aging population, we investigated whether the percentage of diploid cells observed in the aging population on a given day corresponded to the number of cells that remained viable on that day. We gated the population of diploid cells on the (FL2-A SCH) scatter plot graphs obtained during flow cytometry analysis and checked how their proportion changed with time during CLS. As expected, we observed a decreasing percentage of diploid cells in the aging cell population. However, on the 14th and 21st days of the CLS experiment, the numbers of diploid cells observed were very small and did not match the CLS survival results (compare Figures 1A, 3A). We decided to test the correlation between DNA content and cell survival during the CLS assay using the wild-type haploid strain. Figure 3B shows the decreasing percentage of haploid cells accompanying aging of the haploid population. Surprisingly, we observed a higher fraction of cells with haploid DNA content in the last days of the CLS assay of the haploid strain than the fraction of cells with diploid DNA content in the last days of the CLS assay of the diploid strain. This result contradicted the CLS results obtained for haploid and diploid strains (Figure 3C). It remains unclear why aging diploid cells, lacking their typical DNA content (from *2c* to *4c*), survive better than aging haploid cells with preserved full haploid DNA content (from *1c* to *2c*).

Is it possible that a single copy of the yeast genome is enough for survival? To investigate this possibility, we changed the gating parameters and repeated the analysis, as shown in Figure 3D. We observed that not all diploids with typical DNA content were viable. After 4 days, 90% of cells had the expected DNA content, but only 30% were alive. After 7 days, only 30% had the expected DNA content, and 10% were alive. However, on the 14th and 21st day, the proportions were reversed: there were practically no diploids in the assayed population, and about 5% of the cells were still alive. These results can be explained by the fact that one copy of the genome, i.e., *1c*, with all essential genes present, is sufficient for a diploid yeast cell to survive. It appears that diploids can rebuild a second genomic copy based on this single copy because the strains that regrew from the aging population displayed the DNA content typical for diploids (from *2c* to *4c*, Figure 2). These findings suggest that a single copy of the genome can permit the survival of not only haploid but also diploid cells.

This assumption prompted us to reevaluate the flow cytometry data we obtained for the aging cell population during the CLS assay. This time, we compared the DNA content in the whole cell population to the subpopulations gated with respect to different DNA content in the cell. Figure 3E shows the DNA profiles in the subpopulations of aging cells. The systematic decrease of diploids with time was accompanied by an increasing number of cells with lower DNA content. The number of cells with lower than *1c* DNA content better reflect cell mortality than those with DNA content lower than *2c*. This result supports the conclusion that *1c* DNA content permits cell survival during chronological aging.

### Autophagy is induced in the aging cells

The longevity of post-mitotic cells depends on cellular processes capable of degrading old components and replacing them with new ones. According to this scenario, amino acid homeostasis, based on amino acid uptake and recycling, mainly via autophagy of mitochondria and other cellular components, is a process that contributes to the maintenance of cellular homeostasis by removing damaged structures (reviewed in [37]). However, damage also affects other molecules. The loss of proteostasis control and subsequent proteotoxic stress is a well-known hallmark of aging [38]. The damage observed during aging affects all types of molecules, including proteins, lipids, and nucleic acids. Since we detected DNA fragmentation and a gradual decrease in DNA content during the CLS assay, we investigated whether autophagy could be used to clear fragmented DNA.

There are two main pathways involved in the degradation of DNA by autophagy in yeast: nucleophagy and piecemeal microautophagy of the nucleus (PMN) [39]. Each pathway requires a specific protein with a receptor-like function. For nucleophagy, this role is played by Atg39, while for PMN, there are two receptors, Nvj1 and Vac8 [39]. In our experiments, we used cells carrying fluorescently tagged Atg39-GFP (YTE20), Nvj1-GFP (YTE18), Vac8-GFP (YTE19), and GFP-Atg8 (YTE17) as a general control of autophagy induction. The Atg8 protein is activated and overproduced in different autophagy pathways, including the Cvt pathway (i.e., cytoplasm-to-vacuole targeting pathway, which uses autophagosomal-like vesicles for selective transport of proteins to the vacuole), nucleophagy, PMN, mitophagy, and pexophagy. Thus, Atg8 serves as the marker for both micro- and macro-autophagy [40]. Autophagy receptor levels increase during autophagy induction [41–43]. Their levels also increase in stressful conditions known to stimulate autophagy, such as starvation, osmotic, oxidative, or proteotoxic stress [44–46].

**Figure 4:**
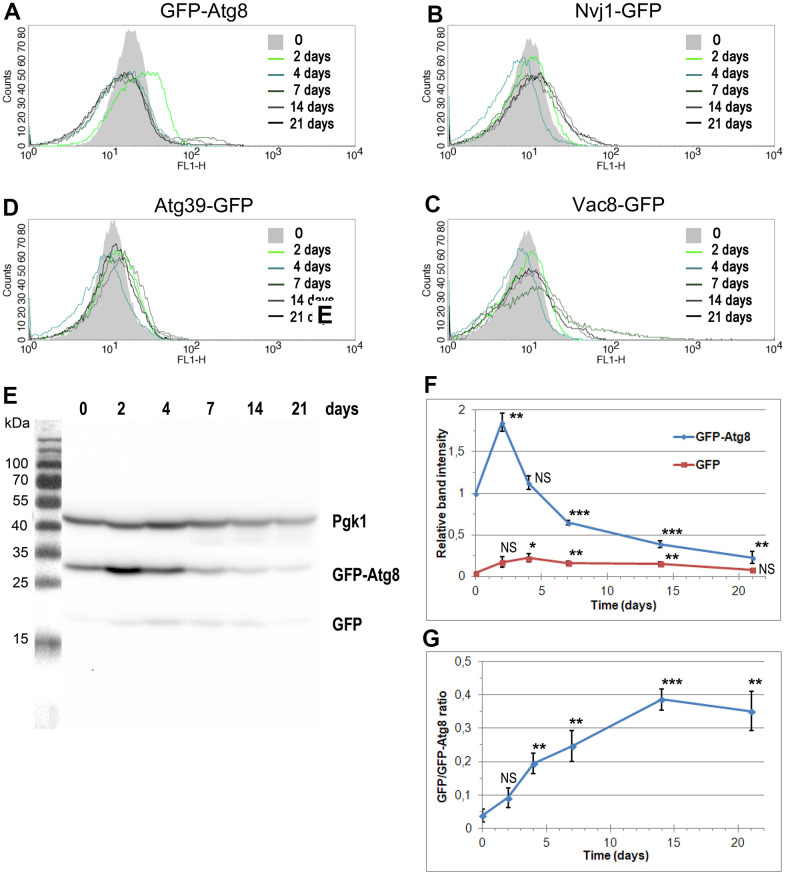
**The changes in the level of autophagy markers during CLS assay.** (**A**–**D**) The level of autophagy markers in the strains carrying fluorescently tagged GFP-Atg8 (YTE17) (**A**), Nvj1-GFP (YTE18) (**B**), Vac8-GFP (YTE19) (**C**), and Atg39-GFP (YTE20) (**D**) measured by flow cytometry. The mean from three independent biological repetitions is shown. (**E**) The level of GFP-Atg8 and its degradation product, free GFP, in the YTE17 strain, visualized by immunoblotting with anti-GFP antibody. One of three independent biological replicates is shown. (**F**, **G**) The quantification of western blot results. (**F**) The level of GFP-Atg8 and its degradation product GFP was normalized to the Pgk1 level and expressed relative to the GFP-Atg8 level at time 0 (protein level in the exponential growth phase). (**G**) The graph shows the GFP/GFP-Atg8 ratio at various time points. Data for each time point was normalized to the Pgk1 level. The mean of three biological replicates is shown for each time point. Bars indicate standard deviations. Statistical significance with respect to time 0 was assessed using the Student T-test (* *p* < 0.05; ** *p* < 0.01; *** *p* < 0.001: NS - non-significant).

To assess the level of autophagy induction, we analyzed the cellular level of several fluorescently tagged proteins, selected as autophagy markers, using flow cytometry. This approach facilitates analysis, especially when proteins of interest are anchored to membranes or possess transmembrane domains, which is the case for PMN and nucleophagy markers. Figure 4A–4D show the results of this analysis. We detected an increase in the fluorescent signal for all tested proteins; however, the pattern of detected changes varied. At the beginning of the CLS experiment, the fluorescent signals fluctuated similarly. Cells expressing Nvj1-GFP, Vac8-GFP, and Atg39-GFP showed a slight increase in green fluorescence on the 2nd day, while on the 4th day, fluorescence decreased, to rise again on the 7th day. However, the scenarios were different for each of the analyzed proteins. In the case of Nvj1-GFP, fluorescence remained at the same level until the end of the CLS experiment. In the case of Atg39-GFP, fluorescence reached a maximum on the 14th day, decreasing later on. In the case of Vac8-GFP, we detected increasing fluorescence on the 7th and 14th day, with a decrease at the end of the CLS experiment. Additionally, the shape of the curve on the histogram changed, suggesting the presence of a subpopulation of cells with very high green fluorescence. These cells were most abundant on the 7th day of the CLS experiment.

The most significant change we observed while analyzing the fluorescence of GFP-Atg8 (Figure 4A). The histogram obtained for this protein shifted to the right on the 2nd day of the CLS experiment, indicating an increased Atg8 level at this time point in the whole cell population. On the 4th day, the level of Atg8 went back to an average level. However, on the 7th day of the CLS experiment, a subpopulation of cells with higher fluorescence appeared, persisting, albeit to a lesser extent, in the following days.

In parallel to the flow cytometry analysis, autophagy activity was measured with the GFP-Atg8 processing assay. Atg8 is efficiently degraded within the vacuole, but GFP is known to be resistant to proteolysis [47]. Subsequently, remains intact for longer, so it can be detected by Western blot and used to indicate nonselective autophagy [48]. Actually, we performed the biochemical assay to monitor both the changes in the Atg8 level and the release of free GFP from GFP-Atg8 in the population of aging cells in time. In Figure 4E–4G, the western blot analysis results are presented. Data showed a significant increase in the GFP-Atg8 level on the 2nd day of the CLS experiment (Figure 4F). We also noticed increasing with time GFP/GFP-Atg8 ratio, indicating the induction of autophagy in the analyzed cells (Figure 4G).

### Nucleophagy and PMN are activated at different time points during chronological aging

Fluorescent tagging of proteins enables the tracking of changes in their cellular distribution. This technique has also been used to study autophagy induction. In the yeast species *S. cerevisiae*, autophagosome formation occurs at the phagophore assembly site (PAS), which is located adjacent to the vacuole [49]. Many of the Atg proteins assemble at the PAS to initiate phagophore nucleation [50, 51]. Since most of the proteins involved in autophagy pathways are transiently associated with the PAS, it is possible to use fluorescence microscopy to assess their fluorescently tagged conjugates, which are localized in proximity to or already within a vacuole [40].

**Figure 5:**
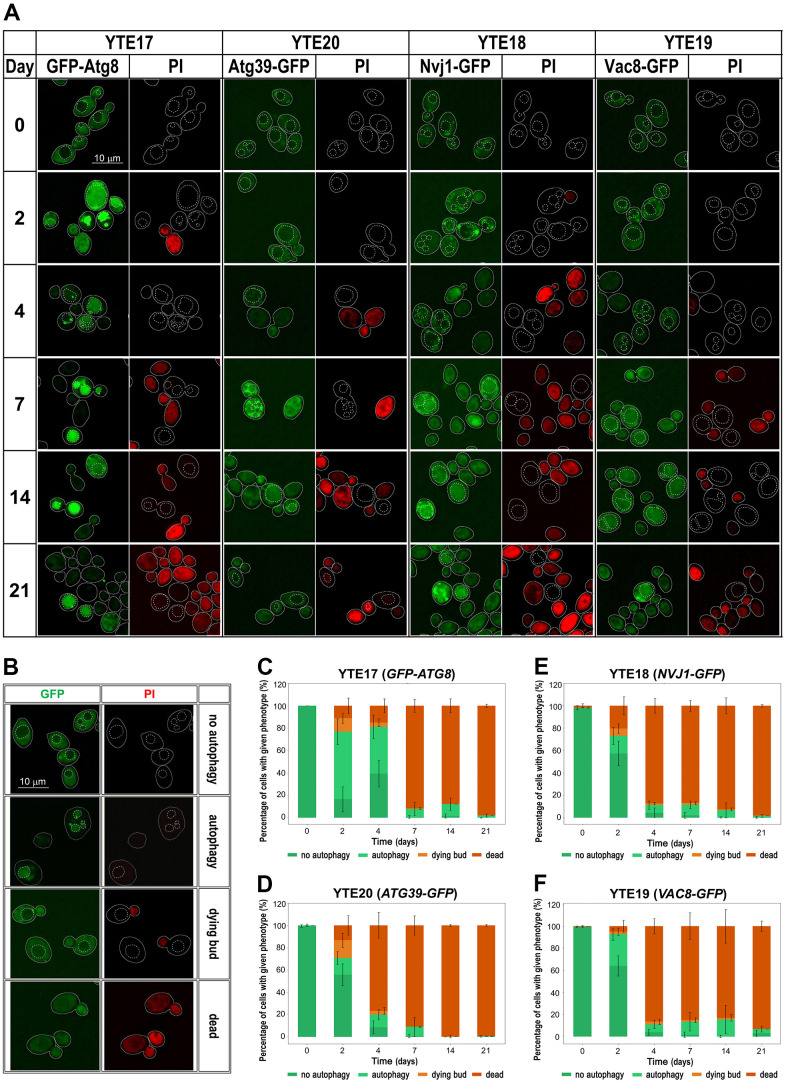
**Changes in the autophagy markers’ localization during chronological aging.** (**A**) Microscopic analysis of strains carrying fluorescently tagged autophagy markers: YTE17 (*GFP-ATG8*), YTE20 (*ATG39-GFP*), YTE18 (*NVJ1-GFP*), and YTE19 (*VAC8-GFP*) in a given day of CLS experiment. Examples shown in (**A**) are focused on cells that were still viable at the analyzed time point. The microscopic images were collected and processed under the same conditions, so the differences in signal intensity are veritable. Cell borders are marked with solid lines, and vacuole outlines, determined based on CMAC staining, are marked with a dashed line. (**B**) Categories of counted phenotypes - examples. (**C**–**F**) The microscopic results quantification is shown as a percentage of cells presenting a given phenotype in the population from a specific time point. Three biological repetitions were performed; in each, at least 300 cells were analyzed for every time point. Graphs show the mean of all biological repetitions; whiskers represent standard deviations. Results obtained for YTE17 (*GFP-ATG8*) (**C**), YTE20 (*ATG39-GFP*) (**D**), YTE18 (*NVJ1-GFP*) (**E**), and YTE19 (*VAC8-GFP*) (**F**) are shown.

We used cells carrying selected fluorescently tagged autophagy markers (GFP-Atg8, Nvj1-GFP, Vac8-GFP, and Atg39-GFP) to track changes in their cellular levels and localization and to correlate their presence in the cell with the viability of tested cells. To indicate the vacuole location in the cell, we used the CellTracker Blue CMAC dye as a vacuole lumen marker. To distinguish between live and dead cells, we used PI staining. Fluorescence microscopy revealed changes in the localization of fluorescently tagged autophagy markers throughout the CLS experiment, suggesting the involvement of different autophagy pathways in aging. However, these pathways were used differently over time. We observed different kinetics of signal recruitment to vacuole contact sites and different times of signal internalization into the vacuole. Some signals also showed a temporary increase in intensity. The most visible increase in signal intensity was observed for GFP-Atg8 on the second day of the CLS experiment, which is consistent with flow cytometry and Western blot data (Figures 4A, 4E, 5A). As a marker of ongoing autophagy, GFP-Atg8 showed obvious phenotypes with sequential changes in signal patterns. First, an increase in the cellular level of the protein was observed, followed by its accumulation in the vicinity of the vacuole, with the PAS contact sites visible as very bright dots. Finally, the protein internalization was visible as an intense bright signal dispersed across the whole vacuole, which decreased with time. These sequential changes were detectable throughout the experiment, but their frequency changed over time (Figure 5A, 5C). The disappearance of the green signal was accompanied by an increase in the red signal of PI-stained cells, indicating cell death.

As shown in Figure 5 (see Supplementary Figure 2 for single-channel images), autophagy induction occurred in all analyzed strains (YTE18: *NVJ1-GFP*, YTE19: *VAC8-GFP*, and YTE20: *ATG39-GFP*), indicating that cells employ both nucleophagy and PMN during chronological aging. However, nucleophagy seems to be particularly engaged in the first few days. We observed Atg39-GFP internalization in a fraction of cells on the 2nd, 4th, and 7th day of the CLS experiment. In contrast, the PMN receptors appeared to be activated throughout the entire experiment. We concluded that nucleophagy is responsible for the initial drop in cell viability, while PMN contributes to cell survival until the end of the CLS experiment. These results are consistent with the findings of fluorescence microscopy studies.

**Figure 6:**
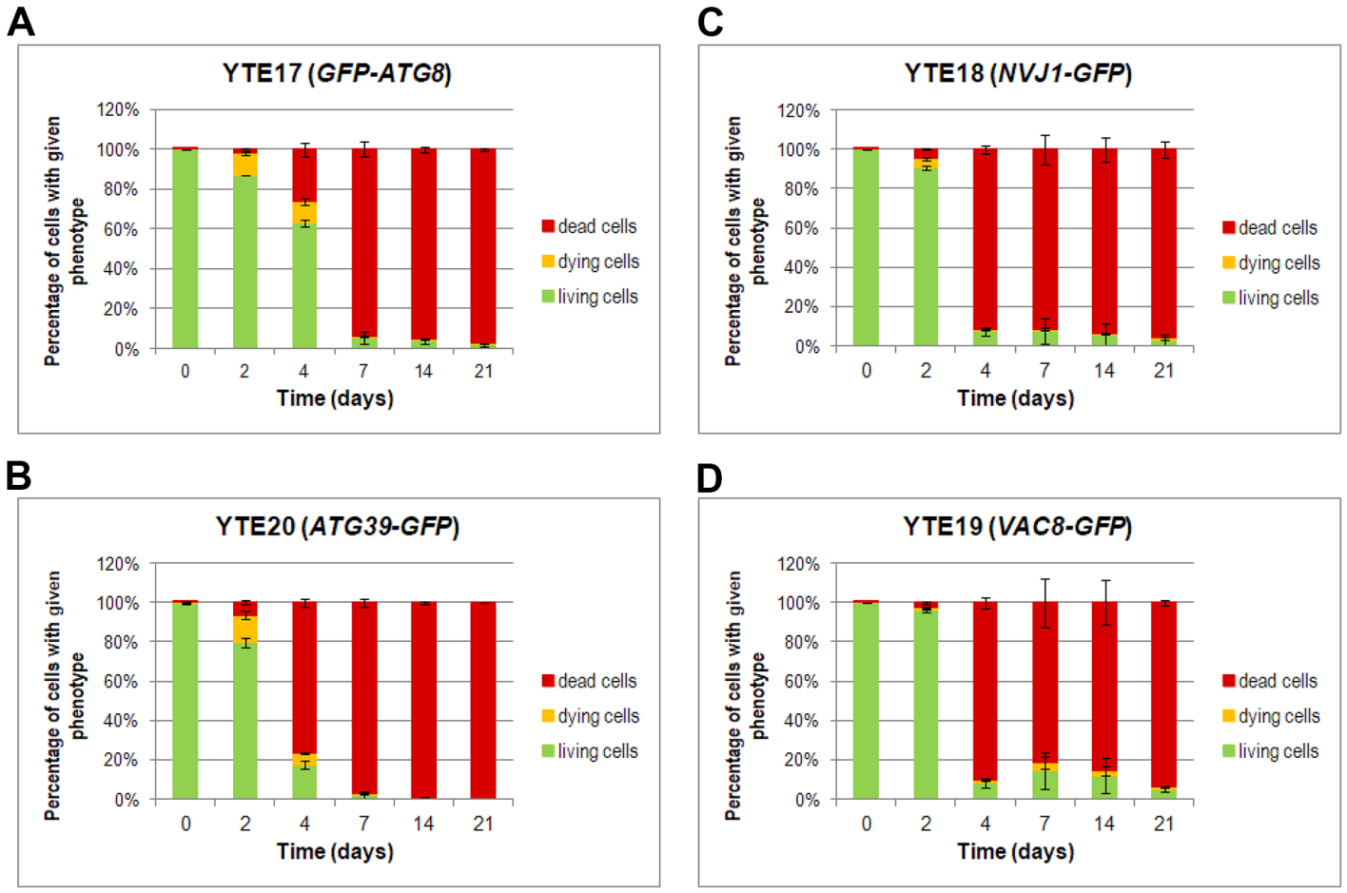
**Changes in the cell viability during CLS, measured by flow cytometry.** Cells of YTE17 (*GFP-ATG8*), YTE20 (*ATG39-GFP*), YTE18 (*NVJ1-GFP*), and YTE19 (*VAC8-GFP*) strains were stained with PI, and fluorescence signals were analyzed in two channels: FL1-H (for GFP) and FL2-H (for PI-stained cells) by flow cytometry in each day of the CLS experiment. According to the presented fluorescence signal, cells in the population were divided into three subpopulations: living, dying, and dead. See Supplementary Material Figures for gating conditions and histograms for each channel for strains and time points. (**A**–**D**) The flow cytometry results quantification was shown as a percentage of cells presenting a given phenotype in the population at a specific time point. Three biological repetitions were performed; and 10,000 events were analyzed for every strain and time point. Graphs show the mean of all biological repetitions; whiskers represent standard deviations. Results obtained for YTE17 (*GFP-ATG8*) (**A**), YTE20 (*ATG39-GFP*) (**B**), YTE18 (*NVJ1-GFP*) (**C**), and YTE19 (*VAC8-GFP*) (**D**) are shown.

Furthermore, a detailed analysis of flow cytometry results provided additional findings on characterizing dying cells. We prepared histograms for individual subpopulations of living, dying, and dead cells defined as cells showing only green fluorescence (cells with GFP signal), showing high green and light red fluorescence (cells with an elevated level of GFP and limited ability to remove PI), and those with strong red fluorescence (cells stained by PI), respectively (Supplementary Figures 3–6). The histograms were prepared for each tested strain (YTE17: *GFP-ATG8*, YTE20: *ATG39-GFP*, YTE18: *NVJ1-GFP*, and YTE19: *VAC8-GFP*) at different time points during chronological aging (Figure 6). These histograms revealed that among cells with induced autophagy, those more prone to death were larger cells with buds. This was particularly evident on the 2nd and 4th days of the CLS experiment in the YTE20 (*ATG39-GFP*) strain population, and from the 2nd day until at least the 7th day of the CLS experiment in the strains carrying fluorescently tagged PMN markers, YTE18 (*NVJ1-GFP*) and YTE19 (*VAC8-GFP*).

Interestingly, for the latter strains’ populations, the histograms that reflect the cell size of living cell subpopulations changed to a bimodal distribution on the last two time points (14 and 21 days). In the histograms of YTE17 (*GFP-ATG8*) strains, the overrepresentation of budding cells among the dying cell population was visible only at the start of the CLS experiment (on the 2nd day). These data correlate with our former observations from fluorescence microscopy, where viable budding mother cells with dying buds were noticed (Figure 5A).

## DISCUSSION

The aging process affects many aspects of life, including at the cellular level, where it pertains to various molecules, such as DNA. As we age, there is an increase in cellular stress, which eventually affects DNA integrity [14, 15, 52]. In this study, we demonstrated that DNA undergoes extensive fragmentation during chronological aging. However, our earlier studies have shown that even highly fragmented DNA can be effectively rebuilt if the DNA repair pathways dedicated to its repair are available [53]. Nevertheless, all DNA damage, including the especially hazardous double-strand breaks (DSBs) that can compromise genome stability, must be repaired for cell survival. At this point, the non-homologous end joining (NHEJ) repair pathway is frequently involved, generating a highly rearranged chromosome where some sequences may even be lost. In such cases, the repaired genome becomes shorter, but the cell will survive as long as the lost fragments do not contain essential genes or sequences crucial for DNA maintenance processes.

Interestingly, a similar observation was reported recently for replicative aging. Mojumdar et al. [54] showed that during replicative aging, resection of DSBs (an early step of DSBs repair via homologous recombination) decreases, while at the same time, the usage of the other pathway of DSBs repair, NHEJ, increases. This results in an increased number of improper products of DSBs repair containing deletions and microhomologies. In chronologically aging cells, endogenously occurring DSBs seem to be defectively repaired, and the frequency of repair defects increases with time, likely leading to senescence [55].

In our studies on chronological aging, we observed a gradual depletion of DNA content in the population of analyzed diploid wild-type cells over time [36]. This result led us to question the mechanism of DNA disappearance and the minimum DNA content required for cell survival. We hypothesized that autophagy could be a probable cause of this phenomenon based on two reasons. First, at least two specialized autophagy pathways, namely nucleophagy and PMN, have been shown to be involved in the processing of nuclear content, including its DNA content [39, 56, 57]. Second, autophagy has been demonstrated to be involved in the chronological aging process [32].

Our data showed that nucleophagy and PMN are indeed activated during chronological aging but at different time points in the CLS experiment. The nucleophagy pathway was activated on the 2nd and 4th days of the CLS experiment, correlating with the steep decline in cell viability detected in the first days of the CLS assay. On the other hand, the PMN pathway was active from the 4th day until the end of the CLS experiment. We concluded that nucleophagy leads to cell death, while PMN contributes to cell survival, likely by removing potentially recombinogenic DNA fragments from the nucleus, where they may provoke genome rearrangements. However, this piece-by-piece removal of genomic DNA may not last indefinitely, as there is a minimum DNA content required for life. According to our results, the minimal level of DNA allowing the survival of diploid cells is one copy of the genome, i.e., *1c* DNA content.

Our CLS studies revealed an interesting observation that suggests that budding cells are more prone to death than mother cells. During the CLS experiment, we detected many cells whose buds were significantly stained with PI, while the mother cells were still vivid. We also observed cells with significantly higher GFP fluorescence in the bud than in the mother cells, as well as very small cells stained with PI but remarkably smaller than the average yeast cells. One of the possible explanations would be that budding cells, when trapped by starvation during chronological aging, might abandon mitosis and withdraw necessary compounds from the bud or even cut off the bud just to survive. However, proving such a hypothesis requires further investigation. This hypothesis contradicts the previously propagated belief concerning the relationship between mother and daughter cells, which claimed that mother cells would sacrifice themselves for the benefit of daughter cells, at least in the context of replicative aging rules [58–60]. However, this is not a general rule of life. For example, it has been shown that malnutrition, such as a lack of vitamin E, may lead to fetal resorption in pregnant animals, including humans. Thus, the hypothesis that follows the evolutionary idea of abandoning reproduction in limited nutritional resources is not so easily dismissed [61].

Recent research by Irvali et al. [62] suggests that yeast cells may indeed change their decision concerning starting a new replication round when nutrients are depleted after they have already passed the G1/S transition checkpoint (the START point of the cell cycle). The expression of G1/S transition genes depends on SBF (Swi4-Swi6) and MBF (Mbo1-Swi6) transcription activators and Whi5 repressor, and the nuclear localization of these transcription factors is regulated by phosphorylation. Dephosphorylated Whi5 can be re-imported into the nucleus, where it binds its target promoters until, upon replenishment of nutrients, CDK re-activation occurs, which releases transcription of G1/S transition genes [62].

What happens when tight control of the START point of the cell cycle is not possible? The answer is provided by experiments in which Krol et al. describe the phenotypes of *swi6Δ* cells [63]. These cells lack functional G1/S transition genes transcription activator, and permanent replication stress leads to the formation of DSBs. The error-prone DNA repair pathway is then used, such as illegitimate recombination, leading to DNA content alterations, including aneuploidy, and resulting in high cell mortality [63]. Interestingly, under starvation conditions, budding yeast can be efficiently returned to the G1 phase in an autophagy-dependent manner. According to Matsui et al., autophagy prevents aberrant nuclear division that occurs despite insufficient cell growth, which can lead to an increased frequency of aneuploidy after supplementing a missing feed source [64]. Notably, we observed an overrepresentation of cells in the G1 phase on the 2nd and 4th day of the CLS assay (Figure 3E).

One more point should be taken into account when considering the mortality of chronologically aging cells: during starvation, yeast cells release autotoxins. It has been shown for many yeast species, including *Schizosaccharomyces pombe*, that cells release toxic compounds such as leucic acid and L-2-keto-3-methyl valerate during glucose depletion, which can kill sister cells [65]. A similar phenomenon of self-inhibition was noticed in *S. cerevisiae* aerobic fed-batch cultures [66]. The results showed that the growth decline observed in those cultures, even when maintained under optimal conditions, should be attributed to self-produced inhibitory compounds other than ethanol, likely cellular metabolites and toxic by-products [67]. These toxic compounds might be particularly harmful to cells with stress-induced transient membrane permeability [68]. It is worth mentioning that the transition in membrane permeability is also observed during autophagy.

In recent years, much new data has emerged concerning the timing and protein-specificity dependence of various autophagy pathways, including nucleophagy and PMN, leading to some disorientation in the field. Receptor-like proteins such as Nvj1 and Vac8 were frequently used as suitable markers to follow PMN, and Atg39 was considered recommended for following nucleophagy [39, 56, 69]. However, recent *in vivo* data showed that the autophagy subpathways are not as specific and separable as previously thought. The same protein might contribute to both PMN and nucleophagy, as demonstrated in the context of Atg39 [67]. Additionally, the researchers’ notion concerning the sequence of events that accompany the autophagy process appears not to be definitive. Mijaljica et al. previously showed that in nitrogen-starved, wild-type haploid yeast cells, PMN and so-called “late nucleophagy” are distinct in time, with PMN starting 3 h after acquiring starvation conditions, whereas nucleophagy can be detected only after prolonged periods of nitrogen starvation (after at least one day) [69]. However, our data suggested different scenarios, with nucleophagy starting first, followed by PMN.

Nevertheless, we must remember that the experimental conditions we used were different in many respects. We analyzed diploid cells for a much longer period, starting from the second day of starvation, under conditions typical of the CLS experiments. Our experimental model was tailored to the questions we asked, which focused on the fate of DNA during chronological aging.

What would be the role of autophagy pathways during chronological aging in the context of cellular genome fate? We believe that the autophagy subpathways activated during chronological aging are crucial in (1) eliminating from the aging population cells that underwent aberrant mitosis; (2) preventing further entry into cell cycle phases, which prevents division abnormalities expected during starvation conditions; and (3) excluding from the nucleus potentially recombinogenic DNA fragments that may lead to DNA rearrangements.

One more issue should be addressed here, namely the puzzling result of the regrowth experiment. The way the regrowth experiment is performed excludes the possibility the regrown population is an offspring of diploids that survived the CLS experiment since the fraction of diploids at the end of the CLS experiment is too small to reach the observed density in the regrown cells population. We considered mating as a diploid source in the regrown population. However, mating is of low probability in a low-density culture containing a limited number of vivid cells and intensively shaken. The alternative source of diploids would be endoreduplication. We currently believe it is the most probable source of diploids in the regrown population of aged cells, primarily since endoreduplication is known to occur in yeast [70].

## CONCLUSIONS

**Figure 7:**
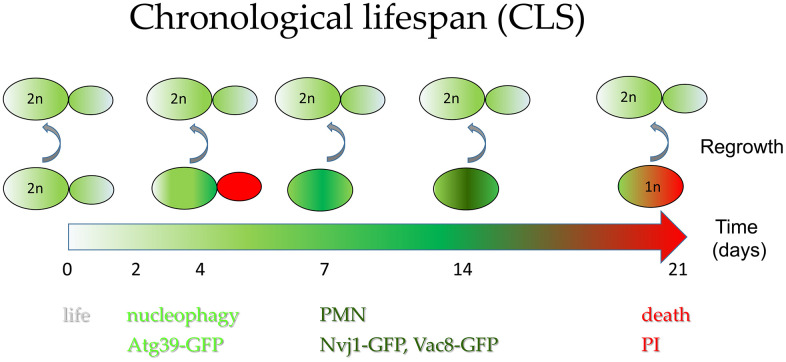
**Autophagy contributes to cells’ survival during chronological aging.** Simplified hypothetical model of diploid cell aging showing (1) activation of nucleophagy at the time of the steepest drop in cells survival during CLS experiment and (2) activation of PMN allowing removal of damaged DNA from the nucleus, likely preventing DNA rearrangements. The unusual phenotype of temporal accumulation of cells with propidium iodide-labeled buds is encompassed in the scheme. This scheme also underlines that cellular DNA content is gradually lost with time during chronological aging, up to the haploid level. Still, only diploid cells can regrow. PMN - piecemeal microautophagy of nucleus; PI - propidium iodide-stained cells.

We found that the chronological aging of diploid yeast *S. cerevisiae* cells is accompanied by extensive DNA fragmentation and a gradual loss of genomic DNA over time, which becomes lethal as soon as the DNA content of a given cell drops below *1c*. The highest decrease in cells’ viability during chronological aging occurs when the nucleophagy pathway is activated, and the cell population is enriched in dying cells with PI-stained buds, with a prevalence of cells in the G1 phase. The further decrease in the chronological aging cells’ DNA content seems dependent on PMN. We believe that this DNA depletion is linked to the clearance from the nucleus of potentially recombinogenic DNA fragments that could provoke DNA rearrangements. Thus, PMN contributes to genome maintenance and promotes survival in starvation conditions during aging (Figure 7). The fragmentation and loss of DNA is a known trait of dying cells also those subject to aging, especially in the context of cell death. The ability of aged cells with reduced ploidy to rebuild the diploid genome during regrowth was not, to our knowledge, documented before. The involvement of autophagy mechanisms in ploidy reduction suggests that it is not a random catastrophic event but a process beneficial to cell survival.

Since yeast cells are widely used as a eukaryotic cell model in aging research studies, we can extrapolate our results to higher eukaryotes. The chromosome fragmentation we documented during chronological aging and the subsequent engagement of autophagic pathways linked to the nucleus do resemble chromoanagenesis in mammals. The observations we made in aging research using yeast as the eukaryotic cell model may help to understand the mechanisms that prevent aneuploidy during aging or cancerogenesis in cells where chromothripsis has occurred.

## MATERIALS AND METHODS

### Yeast strains

The diploid yeast strains used in this study were in the BY474X background and are listed in Table 1. They were the derivatives of the respective haploid strains taken from the library of *S. cerevisiae* strains carrying the genomic GFP-fusions of individual ORFs [71]. In these strains, the mating type was changed using transient expression of Ho endonuclease from the pGAL-HO.Ura plasmid [72]. The resulting *MATα* strains were then crossed with the respective isogenic *MATa* strains to obtain diploid homozygous in respect to the desired gene fusion encoding the selected autophagy marker proteins tagged with GFP. Obtained strains were tested by flow cytometry to confirm their ploidy.

**Table 1:** Strains used in this study.

**Strain**	**Genotype**	**Source**
**BY4741**	*MATa his3∆1 leu2∆0 met15∆0 ura3∆0*	Euroscarf
**BY4743**	*MATa/Matα his3∆1/his3∆1 leu2∆0/leu2∆0 met15∆0/met15∆0 ura3∆0/ura3∆0*	Euroscarf
**BY4741 GFP-ATG8**	*MATa his3∆1 leu2∆0 met15∆0 ura3∆0 LYS2+ can1∆::GAL1pr-SceI::STE2pr-SpHIS5 lyp1∆::STE3pr-LEU2* ***ATG8p-sfGFP-ATG8***	SWAT [70]
**BY4741 NVJ1-GFP**	*MATa his3∆1 leu2∆0 met15∆0 ura3∆0 lys+ can1∆::GAL1pr-SceI::STE2pr-SpHIS5;* ***HPH::NVJ1p-NVJ1-sfGFP***	[70]
**BY4741 ATG39-GFP**	*MATa his3∆1 leu2∆0 met15∆0 ura3∆0 lys+ can1∆::GAL1pr-SceI::STE2pr-SpHIS5;* ***HPH::ATG39p-ATG39-sfGFP***	[70]
**BY4741 VAC8-GFP**	*MATa his3∆1 leu2∆0 met15∆0 ura3∆0 lys+ can1∆::GAL1pr-SceI::STE2pr-SpHIS5;* ***HPH::VAC8p-VAC8-sfGFP***	[70]
**YAS433**	*MATα his3∆1 leu2∆0 met15∆0 ura3∆0 lys+ can1∆::GAL1pr-SceI::STE2pr-SpHIS5 lyp1∆::STE3pr-LEU2* ***ATG8p-sfGFP-ATG8***	this work
**YAS434**	*MATα his3∆1 leu2∆0 met15∆0 ura3∆0 lys+ can1∆::GAL1pr-SceI::STE2pr-SpHIS5;* ***HPH::ATG39p-ATG39-sfGFP***	this work
**YAS435**	*MATα his3∆1 leu2∆0 met15∆0 ura3∆0 lys+ can1∆::GAL1pr-SceI::STE2pr-SpHIS5;* ***HPH::VAC8p-VAC8-sfGFP***	this work
**YAS437**	*MATα his3∆1 leu2∆0 met15∆0 ura3∆0 lys+ can1∆::GAL1pr-SceI::STE2pr-SpHIS5;* ***HPH::NVJ1p-NVJ1-sfGFP***	this work
**YTE17**	*MATa/Matα his3∆1/his3∆1 leu2∆0/leu2∆0 met15∆0/met15∆0 ura3∆0/ura3∆0 lys+can1∆::GAL1pr-SceI::STE2pr-SpHIS5/ can1∆::GAL1pr-SceI::STE2pr-SpHIS5 lyp1∆::STE3pr-LEU2/ lyp1∆::STE3pr-LEU2* ***ATG8p-sfGFP-ATG8/ATG8p-sfGFP-ATG8***	this work
**YTE18**	*MATa/Matα his3∆1/his3∆1 leu2∆0/leu2∆0 met15∆0/met15∆0 ura3∆0/ura3∆0 lys+ can1∆::GAL1pr-SceI::STE2pr-SpHIS5/can1∆::GAL1pr-SceI::STE2pr-SpHIS5;* ***HPH::NVJ1p-NVJ1-sfGFP/HPH::NVJ1p-NVJ1-sfGFP***	this work
**YTE19**	*MATa/Matα his3∆1/his3∆1 leu2∆0/leu2∆0 met15∆0/met15∆0 ura3∆0/ura3∆0 lys+ can1∆::GAL1pr-SceI::STE2pr-SpHIS5/can1∆::GAL1pr-SceI::STE2pr-SpHIS5;* ***HPH::VAC8p-VAC8-sfGFP/HPH::VAC8p-VAC8-sfGFP***	this work
**YTE20**	*MATa/Matα his3∆1/his3∆1 leu2∆0/leu2∆0 met15∆0/met15∆0 ura3∆0/ura3∆0 lys+ can1∆::GAL1pr-SceI::STE2pr-SpHIS5/can1∆::GAL1pr-SceI::STE2pr-SpHIS5;* ***HPH::ATG39p-ATG39-sfGFP/HPH::ATG39p-ATG39-sfGFP***	this work

### Growth conditions and media

Yeast strains were grown in a minimal synthetic medium SCD containing 0.67% (w/v) Yeast nitrogen base without amino acids (Difco, Mt Pritchard, NSW, Australia), 2% (w/v) glucose (POCh, Gliwice, Poland) and amino acids (Formedium, Hunstanton, UK): L-leucine (180 mg/l), L-histidine (60 mg/l), uracil (60 mg/l) and methionine (60 mg/l). Each investigated strain was cultured in 120 ml of liquid medium, in 3 biological repeats. Cells were grown on a shaker at 150 rpm, 28° C for 21 days. At selected time points (0, i.e, exponential phase of growth, 2 days, 4 days, 7 days, 14 days and 21 days) 1×10^7^ cells from each culture were harvested and subjected to further analyses.

In the regrowth experiment YPD medium, containing 1% yeast extract (Difco), 2% bactopeptone (Difco), 2% glucose (POCh) was used. To obtain solid medium, 2% agar (Difco) was added.

### Chronological lifespan (CLS) assays

Chronological lifespan of cells incubated in SCD medium was measured as previously described [36]. Briefly, from the growing culture, samples were removed at the indicated time points to assess the survival within the population. The number of colonies forming units (CFU) was counted for the samples taken after 2, 4, 7, 14, and 21 days of cultivation. The data from at least three independent experiments were averaged. Statistical significance of the results was assessed using ANOVA and Dunnett’s post hoc test.

### Regrowth assay

For the regrowth experiment, the 5 -10 μl of culture from a certain time-point of the CLS experiment was used to inoculate 20 ml of YPD medium. The 5 μl was used on the 2^nd^, 4^th^, and 7^th^ days of CLS, while 10 μl for the 14^th^ and 21^st^ day. The cultures were cultivated with shaking at 28° C for 18 h until they reached the density of about 5 x 10^6^ cells per ml. Then, the cells were used for testing their phenotype, e.g., for DNA content analysis.

### DNA content analysis by flow cytometry

The DNA content of yeast cells was measured by flow cytometry as previously described [21], with some modifications. Briefly, cells grown to exponential phase were fixed with 1 ml of chilled (−20° C) 80% ethanol, and then held at room temperature for at least 2 h. The fixed cells were washed twice using FACS buffer (0.2 M Tris-HCl (Sigma-Aldrich, St. Louis, MO, USA) pH 7.4 and 20 mM EDTA (Merck, Darmstadt, Germany)). To remove the RNA, cells suspensions were incubated in FACS buffer with 1 mg/ml RNase A (Sigma-Aldrich) for 2 h at 37° C. The cells were then washed with 1 x PBS, stained with 100 μl of PI solution (50 μg per ml) in 1 x PBS overnight at 4° C in the dark, and diluted with 900 μl of 1 x PBS. Prior to flow cytometry analysis, the cells were sonicated three times for 10 s in a Branson 2800 ultrasonic bath, to avoid cell clumping. The analysis of the DNA content was performed using a FACSCalibur analyzer (Becton-Dickinson, Franklin Lakes, NJ, USA). A total of 10,000 cells in each sample were counted. At least three biological repetitions were assayed. Further data analysis was performed using CellQuestPro software (Becton-Dickinson).

### The genome integrity analysis by electrophoretic methods

To assess yeast chromosome integrity in the aging cells, we separated yeast chromosomes by PFGE. The experiment was performed according to [63] with some modifications. Yeast cells of diploid BY4743 strain, from three biological repeats, were embedded in low-melting-point InCert agarose (Lonza, Basel, Switzerland), in 20 μl plugs and digested with Zymolyase 100T (BioShop, Burlington, ON, Canada), then with proteinase K (Sigma-Aldrich) and RNase A at 30° C with gentle rotation (4 r.p.m.) on an SB3 rotator (Bibby Sterlin, Stone, UK). Plugs were placed in the wells of a gel prepared from 1.2% D5 agarose (Conda, Torrejon de Ardoz, Madrid, Spain) in 1× TAE and sealed with the same agarose. Electrophoresis was run in a CHEF Mapper® XA Pulsed-Field Electrophoresis System (Bio-Rad, Hercules, CA, USA) for 15 h in 1× TAE buffer at 6 V/cm and 12° C with the ramping set to 0.8, the angle set to 120°, and the switch time set to 65–90 s. After electrophoresis, the DNA was stained with SYBR™ Gold Nucleic Acid Gel Stain (Invitrogen, Carlsbad, CA, USA) for 30 min with gentle rocking, washed twice with water for 15 min, and documented by using 302-nm UV light for DNA visualization and a charge-coupled device camera (FluorChem Q Multi Image III, Alpha Innotech, San Leandro, CA, USA).

Agarose-embedded chromosomal DNA from the same cell samples was also separated in 1% D5 agarose gel using 15 h standard electrophoresis in 1 x TAE buffer at 20 V/cm and 12° C. Gel was stained and documented as above.

### Determination of GFP-Atg8 protein levels and GFP-Atg8 processing assay

The GFP-Atg8 and GFP levels change during CLS was assessed by western blot as in [73] with some modifications. Proteins were extracted from 1×10^8^ cells collected at each time point during CLS using the TCA method, suspended in Laemmli sample buffer supplemented with 1 mM PMSF and cOmplete Protease Inhibitor Cocktail (Roche, Base, Switzerland), and boiled for 5 min. After centrifugation (19,300 g for 2 min), the equal volumes of the protein extracts were separated by SDS-PAGE (10% polyacrylamide gel), and the proteins were transferred onto PVDF membrane (GE Healthcare, Pittsburgh, PA, USA). Blots were blocked for 2 h in 5% (w/v) nonfat dried milk + 5% (w/v) BSA (Sigma-Aldrich) in TBS-T [25mM Tris-HCl pH 7.5, 137 mMNaCl, 27 mM KCl, 0.1% (v/v) Tween-20]. GFP protein was detected with the rabbit polyoclonal antibody anti-GFP (1:2000, Living Colors A.v. peptide antibody, 632377) and goat anti-rabbit IgG conjugated to horseradish peroxidase (HRP) (1:4000, Agilent, Cat# P0448, RRID:AB_2617138). Pgk1was detected by using a mouse anti-Pgk1 antibody (1:10000, Abcam, Cat# ab113687, RRID:AB_10861977), followed by goat anti-mouse IgG conjugated to HRP (1:4000, Agilent, Cat# P0447, RRID:AB_2617137). Immunoreactive proteins on the blots were visualized using chemiluminescent substrates: SuperSignal WestFemto, (Thermo Fisher Scientific Inc., Waltham, MA, USA) and documented with a charge-coupled device camera (FluorChem Q Multi Image III, Alpha Innotech, San Leandro, CA, USA). The resulting bands were quantified by using Image Quant 5.2 (Molecular Dynamics, Inc., Sunnyvale, CA, USA). The GFP-Atg8 and GFP proteins levels were normalized to those of Pgk1. The mean of three biological repetitions were averaged to determine the relative protein levels for each time point of CLS. The GFP/GFP-Atg8 ratio was also calculated. Statistical significance with respect to time 0 was assessed using the Student T-test.

### Autophagy induction studies using fluorescence microscopy

Strains carrying the fluorescently tagged autophagy markers (GFP-Atg8, Atg39-GFP, Nvj1-GFP, and Vac8-GFP) were used together with two fluorescent dyes staining the vacuole (CellTracker™ Blue CMAC Dye, Invitrogen) and dead cells (PI, Calbiochem, San Diego, CA, USA) respectively. We followed the changes in the cellular localization of autophagy markers and the correlation between their presence in the cell and the viability of tested cells. Autophagy induction occurs when autophagy markers co-localize with the vacuole.

Harvested cells of strains assigned for fluorescence microscopy analysis were first stained with PI (final concentration 0.1 mg/ml) and CellTracker™ Blue CMAC Dye (final concentration 50 μM) for 30 minutes at room temperature in the dark. Stained cells were washed three times with 1 x PBS. After washing, pelleted cells were suspended in 30 μl of 1 x PBS. The 3 μl of analyzed cells’ suspension was placed on a microscope slide for microscopic analysis. Imaging was performed using Zeiss AxioCam MRc5 Digital Camera (Zeiss, Oberkochen, Germany), mounted on Zeiss Axio Imager.M2 fluorescence microscope operated by Zeiss Axio Vision 4.8 software using: DIC (for bright field), and 49HE, 63HE, and 38HE filter sets (for CMAC, PI, and GFP, respectively). All images were acquired with 100-fold magnification under immersion oil. Fluorescent microscopy images quantitative analysis was performed using Fiji software, using Cell Counter and DeconvolutionLab2 plugins.

### Flow cytometry measurement of autophagy markers level and cells’ viability during CLS

Strains carrying the fluorescently tagged autophagy markers (GFP-Atg8, Atg39-GFP, Nvj1-GFP, and Vac8-GFP) were analyzed by flow cytometry during the CLS experiment. Half of the culture of the tested strain from the given time point of CLS was stained with 0.1 mg/ml PI (Calbiochem, San Diego, CA, USA) in 1 x PBS for 30 minutes in the dark to assess the cells’ viability. PI stains only dead cells. By analyzing both unstained and stained cells, we followed two parameters: (1) the changes in the autophagy markers’ levels, which are believed to increase during autophagy induction; and (2) the correlation between increased autophagy marker signal and viability of tested cells.

The analysis was performed using a FACSCalibur analyzer (Becton-Dickinson, Franklin Lakes, NJ, USA). A total of 10,000 cells in each sample were counted. At least three biological repetitions were assayed. Farther data analysis was performed using CellQuestPro software (Becton-Dickinson).

### Availability of data and materials

All data generated or analyzed during this study are included in this published article and its Supplementary Information Files.

## Supplementary Material

Supplementary Figures
