# Supplementary material for: Live while the DNA lasts. The role of autophagy in DNA loss and survival of diploid yeast cells during chronological aging
Source: Aging (Albany NY). 2023 Oct 9;15(19):9965–83. doi: 10.18632/aging.205102 (PMC10599738; doi:10.18632/aging.205102)
Supplement: Supplementary Figures [file aging-15-205102-s001.pdf]

SUPPLEMENTARY FIGURES

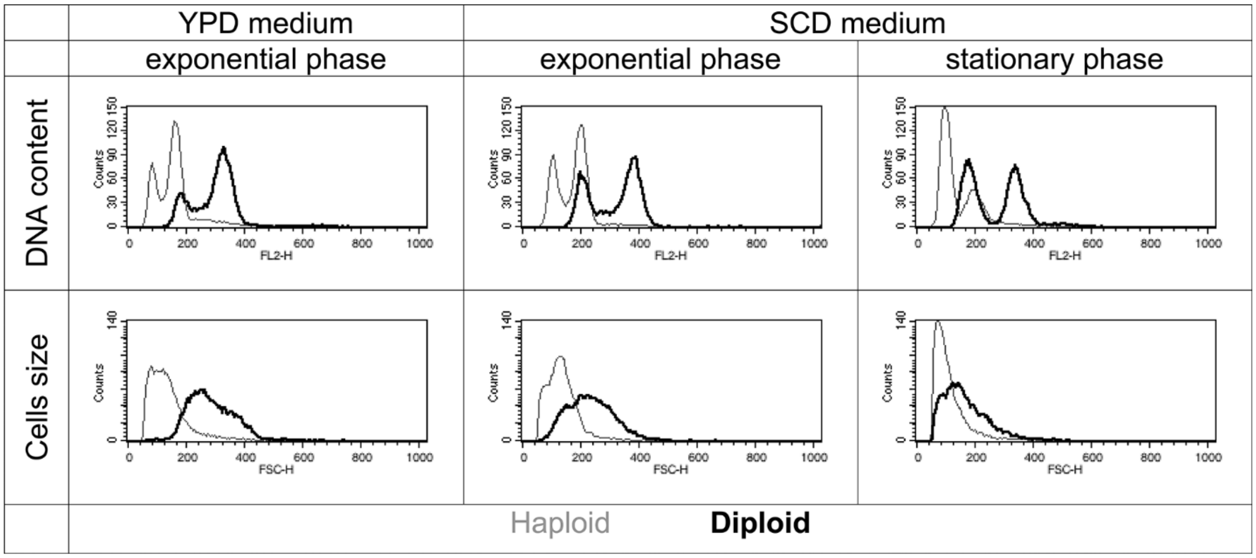

Supplementary Figure 1. DNA content and cells size of control cells cultivated in different type of medium to a given phase of growth. Gray, thin line – Haploid (BY4741); Black, thick line – Diploid (BY4743).

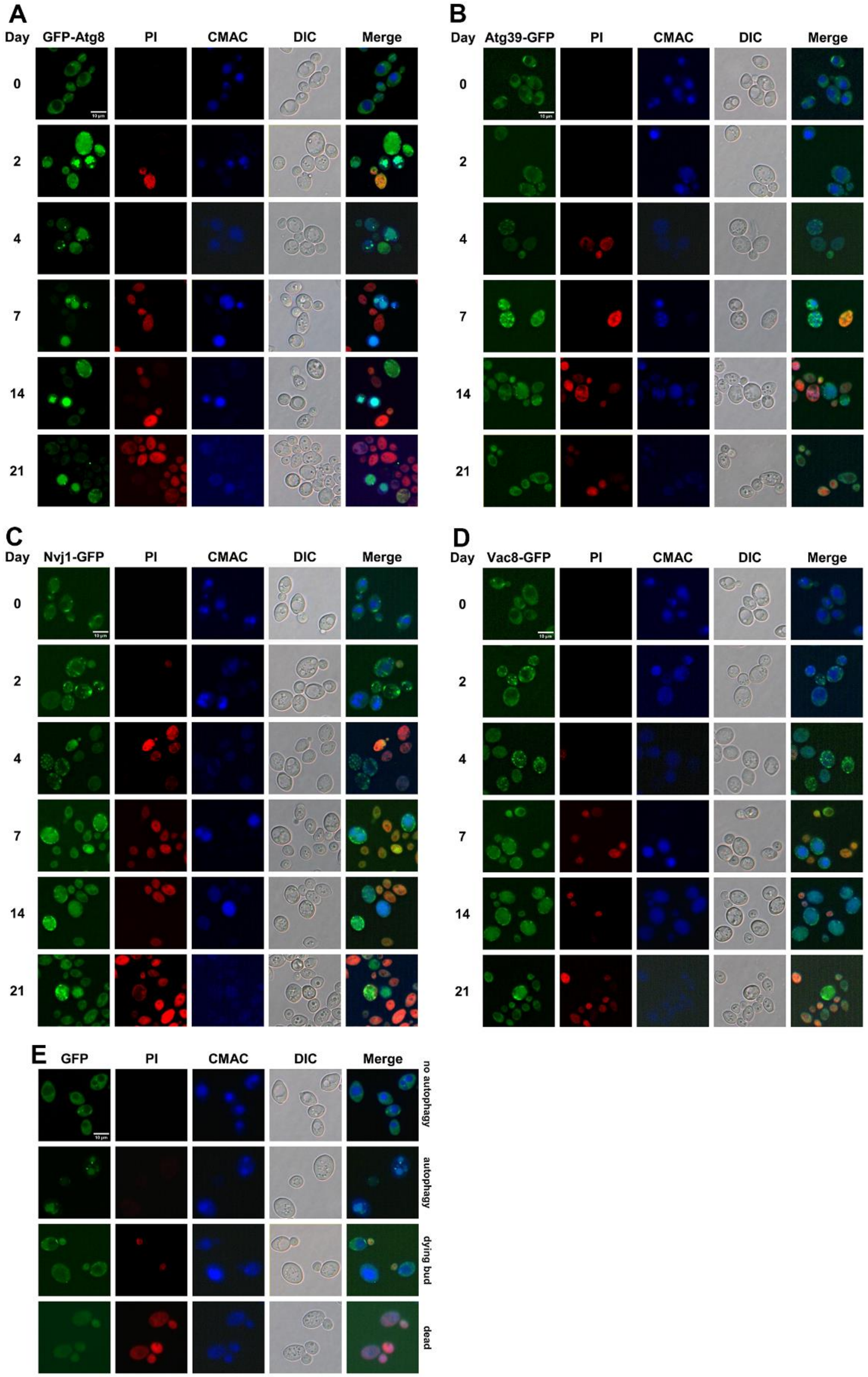

Supplementary Figure 2. Changes in the autophagy markers' localization during chronological aging. Microscopic analysis of strains carrying fluorescently tagged autophagy markers: (**A**) YTE17 (GFP-ATG8), (**B**) YTE20 (ATG39-GFP), (**C**) YTE18 (NVJ1-GFP), and (**D**) YTE19 (VAC8-GFP) in a given day of CLS experiment. Examples shown in (**A**–**D**) are focused on cells that were still viable at the analyzed time point. The microscopic images were collected and processed under the same conditions, so the differences in signal intensity are veritable. (**E**) Categories of counted phenotypes - examples.

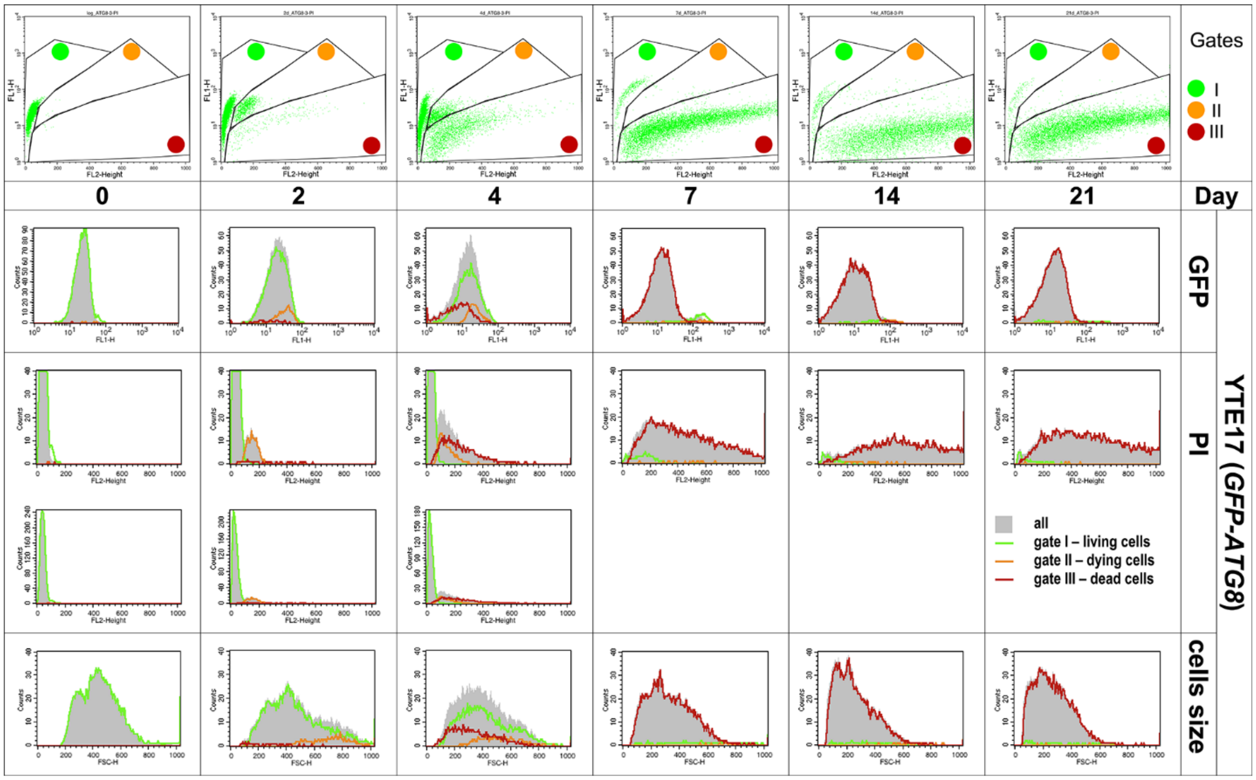

Supplementary Figure 3. Changes in the GFP-Atg8 fluorescence and cell viability measured by flow cytometry during the CLS of YTE17 (GFP-ATG8). Cells in the population were divided into three subpopulations: living, dying, and dead, according to the presented fluorescence signal. The upper panel shows gating conditions. Lower panels show histograms for each channel and time point.

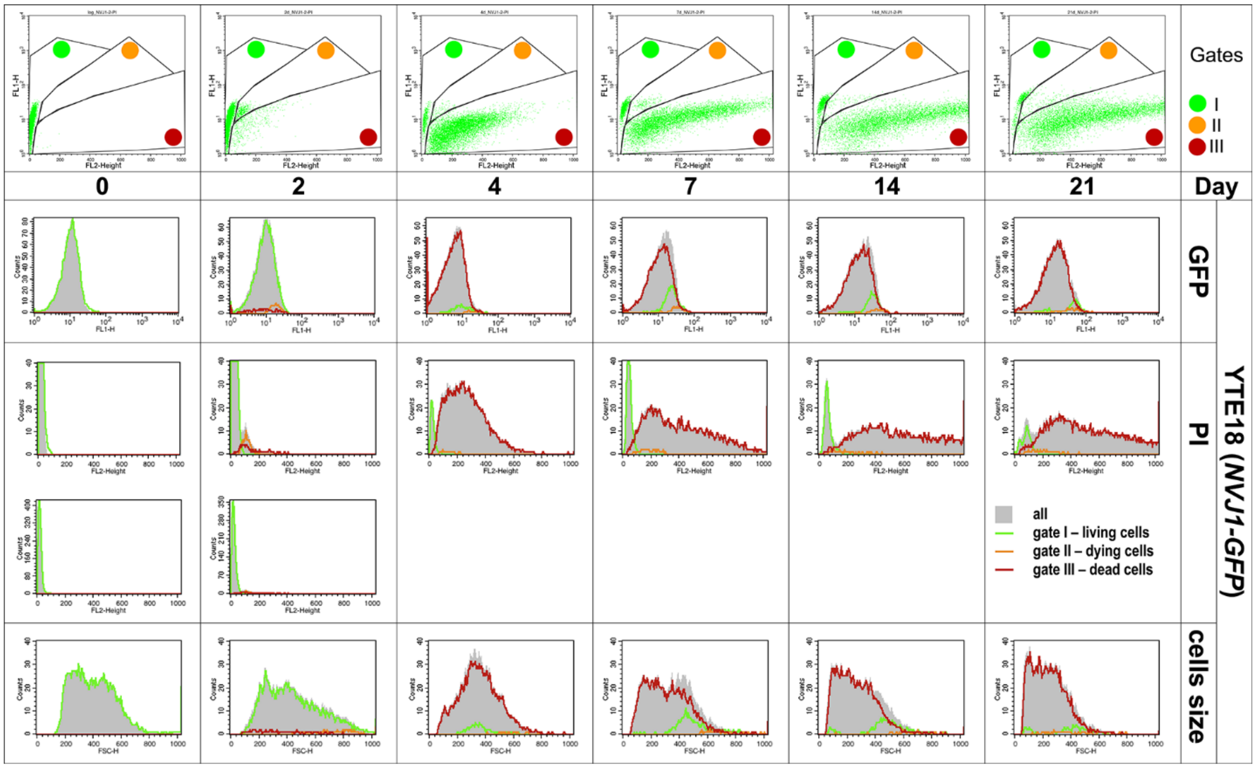

Supplementary Figure 4. Changes in the Nvj1-GFP fluorescence and cell viability measured by flow cytometry during the CLS of YTE18 (NVJ1-GFP). Cells in the population were divided into three subpopulations: living, dying, and dead, according to the presented fluorescence signal. The upper panel shows gating conditions. Lower panels show histograms for each channel and time point.

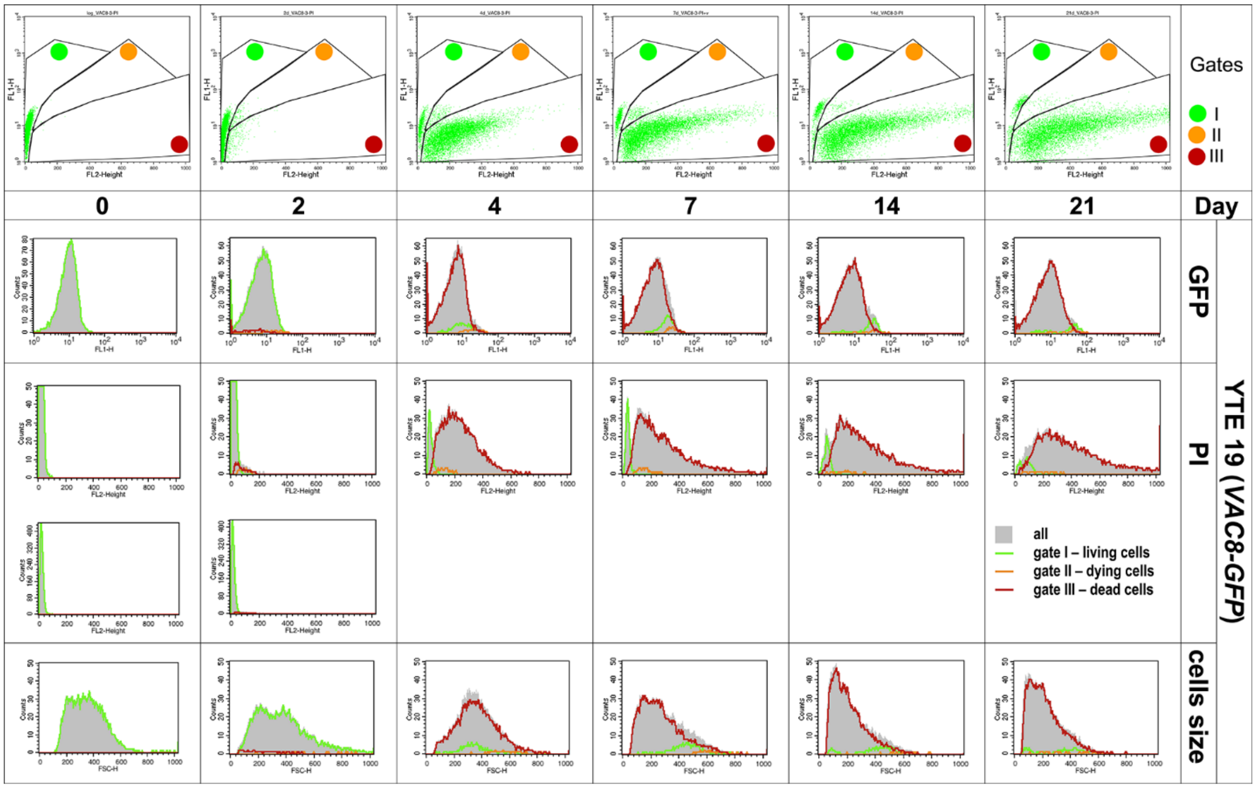

Supplementary Figure 5. Changes in the Vac8-GFP fluorescence and cell viability measured by flow cytometry during the CLS of YTE19 (VAC8-GFP). Cells in the population were divided into three subpopulations: living, dying, and dead, according to the presented fluorescence signal. The upper panel shows gating conditions. Lower panels show histograms for each channel and time point.

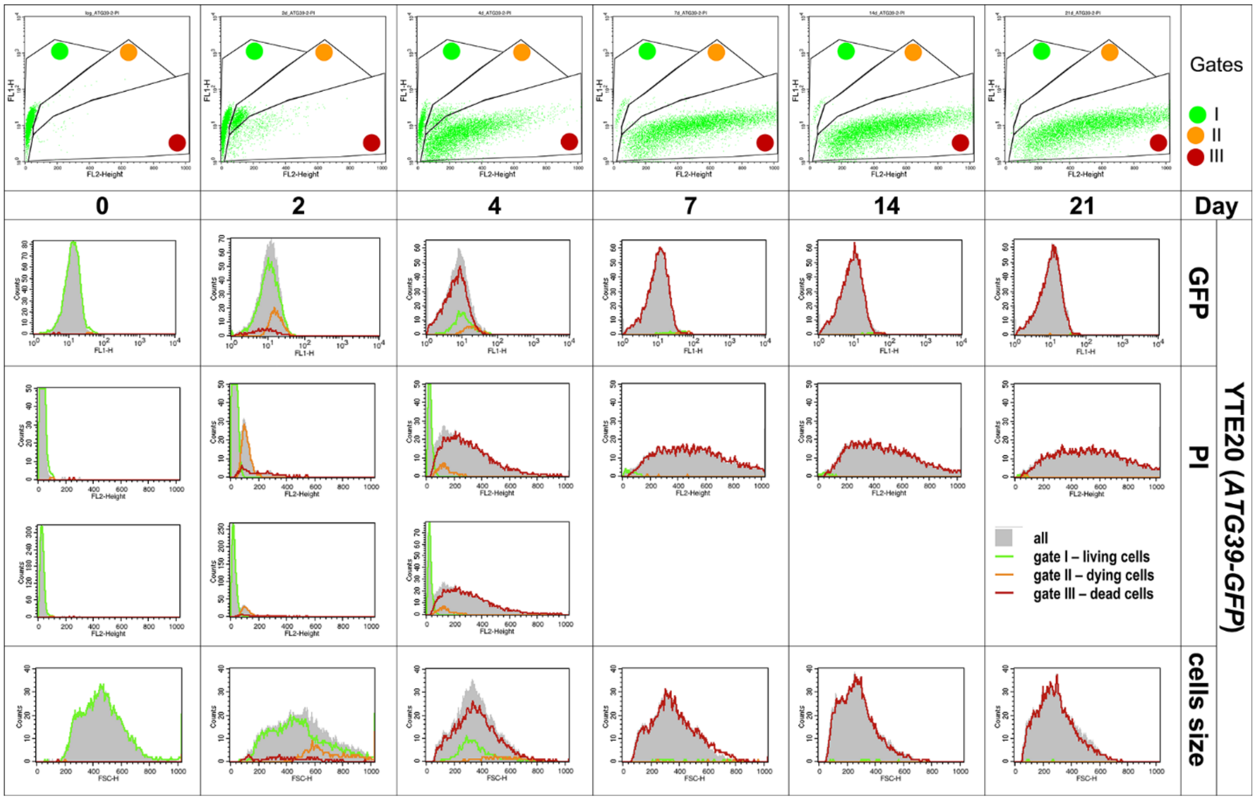

Supplementary Figure 6. Changes in the Atg39-GFP fluorescence and cell viability measured by flow cytometry during the CLS of YTE20 (ATG39-GFP). Cells in the population were divided into three subpopulations: living, dying, and dead, according to the presented fluorescence signal. The upper panel shows gating conditions. Lower panels show histograms for each channel and time point.
